# Resilience among women survivors of intimate partner violence: Coping, meaning-making, and well-being

**DOI:** 10.1017/gmh.2026.10262

**Published:** 2026-06-25

**Authors:** Çiğdem Tini, Halis Sakiz

**Affiliations:** 1 Mardin Directorate of Violence Prevention and Monitoring Centre, Ministry of Family and Social Services, Mardin, Türkiye; 2 Department of Educational Sciences, Faculty of Letters, Mardin Artuklu University, Mardin, Türkiye

**Keywords:** intimate partner violence, women, coping, meaning-making, well-being

## Abstract

This qualitative study explores how women survivors of intimate partner violence (IPV) cope with abuse and reconstruct well-being and meaning in their lives. The study was conducted with 16 women aged between 27 and 51 living in three cities in the southeastern region of Türkiye. Participants were recruited through women’s shelters and non-governmental organizations (NGOs) using purposive and snowball sampling. Data were collected through semi-structured interviews and analyzed using thematic analysis. The findings indicate that women’s experiences of violence were shaped by sociocultural gender norms and included physical, psychological, sexual, and economic abuse. Participants described multiple coping strategies, including seeking social support, emotional expression, religious coping, and resisting stigmatization. Women’s pathways toward well-being involved gaining economic independence, developing healthier communication patterns, and investing in personal development. Meaning-making processes included redefining self-identity, motherhood, reconnecting with positive experiences, and interpreting survival as a source of strength. The findings highlight survivors’ agency and resilience and underline the importance of social, psychological, and economic support mechanisms in facilitating recovery following IPV.

## Graphical abstract

**Figure:**
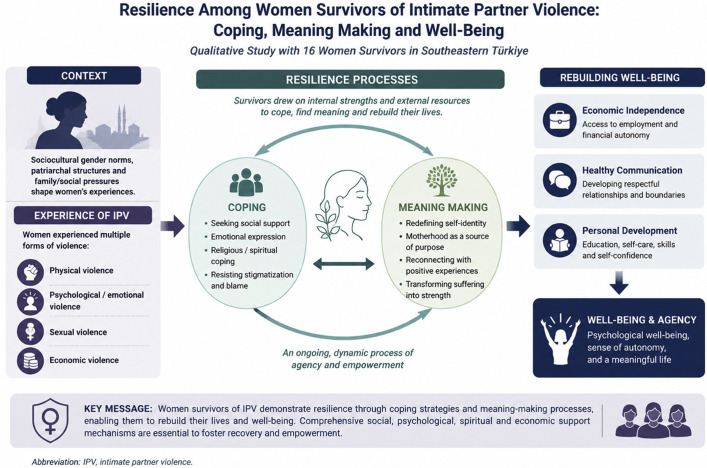

## Impact statement

Intimate partner violence (IPV) is a major public health and human rights concern. However, much research focuses primarily on the harms experienced by survivors. This study highlights the resilience, agency, and meaning-making processes of women who have experienced IPV in the southeastern region of Türkiye. By documenting survivors’ lived experiences, the research shifts the focus from victimization alone toward the complex ways women cope with violence, rebuild their lives, and restore well-being. The findings show that recovery from IPV is a gradual process shaped by social, economic, and cultural factors. Women’s narratives reveal how recognizing violence as a violation of rights can become a turning point that enables help-seeking, resistance, and reclaiming autonomy. Coping strategies, including social support, emotional expression, religious coping, and resistance to stigma, played important roles in recovery, showing resilience as a dynamic process emerging through interactions between personal agency and supportive environments. Access to employment, social networks, psychological services, and supportive community contexts was particularly important in helping women rebuild their lives and regain well-being. These findings highlight the importance of interventions that strengthen economic opportunities, mental health services, and supportive environments for survivors of IPV. The study also provides practical implications for policymakers, social workers, counselors, and organizations working in the field of gender-based violence. By foregrounding survivors’ voices and experiences, it supports the development of more responsive and empowering support systems. Ultimately, the study contributes to broader efforts to challenge gender-based violence, promote women’s rights, and foster social conditions that enable survivors not only to escape violence but also to rebuild meaningful and fulfilling lives.

## Introduction

Violence is a harmful behavior that affects various aspects of life (John et al., 2020; Rollero et al., 2021). Its detrimental effect on quality of life is a major 21st-century concern (Stöckl and Sorenson, 2024). Violence against women is widely recognized as a significant global public health and human rights concern. The World Health Organization (WHO) estimates that approximately one in three women worldwide has experienced physical and/or sexual violence, most often perpetrated by an intimate partner (Sardinha et al., 2022; Stöckl and Sorenson, 2024). While societal mechanisms often address external violence against women, intimate partner violence (IPV) is frequently concealed (Bilican Gökkaya, 2009).

In Türkiye, IPV is a persistent issue (Boyacıoğlu, 2016). Women’s movements since the 1980s, through campaigns and non-governmental organizations (NGOs) like Mor Çatı (1998) and The Foundation for Women’s Solidarity (2017) (Köknel, 1996; Özmen, 2004; Altınay and Arat, 2007), have aimed to address this issue. Despite efforts, a study showed that 40% of Turkish women experience physical violence with high rates of other abuse (Ministry of Family and Social Services and Hacettepe University, 2014). This global health concern violates rights (Cepeda et al., 2022) and has social, economic, and psychological consequences (Altınay and Arat, 2007; Reeves, 2020).

Gender inequality, which stems mostly from social roles (Hester et al., 2025), is a key driver that hinders women’s progress and contributes to harmful practices (Reeves, 2020; Ceylan-Batur et al., 2023). This societal issue requires comprehensive policy and family-centered interventions (Lombardo and Rolandsen Agustín, 2016), given the detrimental effects on women’s health and development (Özmen, 2004; Grose et al., 2021; True, 2021; Nnyombi et al., 2022; Wessells and Kostelny, 2022). Given this pervasive reality, this study explores the lived experiences of women experiencing IPV by focusing on coping mechanisms for ending it and their journey to well-being and meaning-making post-IPV.

## Conceptual framework

### IPV and survivors’ recovery

IPV refers to a pattern of abusive behaviors occurring within an intimate relationship and disproportionately affecting women (Adibelli et al., 2021; Kisa et al., 2023). IPV can manifest in multiple forms, including physical (Balci and Ayranci, 2005), sexual (Li et al., 2023), emotional or psychological (Alkan et al., 2022), and economic violence (Alkan et al., 2021). Physical violence involves the use of force resulting in bodily harm, while sexual violence encompasses non-consensual sexual acts. Psychological violence refers to behaviors that cause emotional distress, humiliation, or fear, and economic violence includes restricting access to financial resources and employment opportunities. These forms of violence frequently co-occur and have profound consequences for women’s physical health, psychological well-being, and social functioning.

IPV is embedded in broad gendered power relations and often involves patterns of coercion and control within intimate relationships. In many sociocultural contexts, including Türkiye, IPV is also shaped by broader family structures and intergenerational gender norms. Family environments may contribute to the normalization of male authority, women’s obedience, and the acceptance or concealment of violence within intimate relationships (Rasool, 2015). Extended family members, including parents, in-laws, and other relatives, may directly or indirectly reinforce abusive dynamics through pressure to maintain marriages, silence disclosure of violence, or prioritize family reputation over women’s safety. Prior exposure to violence within the family may also shape women’s later perceptions of abuse and influence coping responses. Examining IPV within these broader familial and sociocultural contexts is therefore important for understanding both women’s vulnerability to violence and their pathways toward resilience and recovery.

In the southeastern region of Türkiye, women’s experiences of IPV may also be shaped by relatively conservative gender norms, collectivist family structures, and social expectations surrounding family honor and marital continuity. In some communities, women may experience pressure to maintain marriages despite abuse due to concerns about stigma, family reputation, and social exclusion following separation or divorce. Arranged marriages, economic dependency, and limited access to social support services may further constrain women’s options, particularly in more traditional or socioeconomically disadvantaged contexts (Dolkun et al., 2025). At the same time, urbanization, education, and changing social attitudes may create tensions between traditional gender expectations and women’s growing aspirations for autonomy and equality. Understanding these sociocultural dynamics is important for interpreting women’s coping strategies, help-seeking behaviors, and recovery processes following IPV.

Although violence may occur in public and private spheres, IPV often remains hidden within the family and may fail to trigger an adequate social response (Zulver, 2025). Consequently, survivors often experience personal and structural barriers when attempting to cope with abuse and rebuild their lives. These challenges highlight the importance of examining the harms associated with IPV and the processes through which survivors mobilize resources and reconstruct their lives following violence.

### Resilience as a framework for understanding survivors’ adaptation

Resilience is a useful framework for understanding how individuals respond to and recover from adversity. Resilience is generally defined as a dynamic process through which individuals adapt positively despite experiencing significant hardship or trauma (Masten, 2001). Rather than representing a fixed personality trait, resilience reflects ongoing interactions between individuals and their social environments, including personal coping capacities, social support networks, and cultural meanings that shape responses to adversity.

In the context of IPV, resilience does not imply the absence of harm or distress. Instead, it refers to survivors’ capacity to navigate adversity, mobilize internal and external resources, and reconstruct a sense of safety, identity, and well-being after experiences of violence. Research on IPV has increasingly emphasized that survivors are not merely passive victims but active agents who employ diverse strategies to cope with violence, seek support, and rebuild their lives (Hamby et al., 2018). Within this perspective, resilience is understood as an adaptive process that unfolds through mechanisms such as coping and meaning-making and may contribute to the gradual restoration of psychological and social well-being.

### Conceptual relationships among the core constructs

Although coping, resilience, well-being, and meaning-making are closely related, they refer to distinct but interconnected processes within survivors’ recovery after IPV. In this study, resilience is conceptualized as an overarching adaptive process through which women respond to and recover from violence over time. Coping refers to the specific cognitive, emotional, and behavioral strategies women use to manage the immediate and ongoing demands of abuse. Meaning-making refers to survivors’ efforts to interpret their experiences, reconstruct identity, and restore a sense of purpose and coherence following violence. Well-being is treated as a broader outcome of recovery reflected in psychological and social functioning. Post-traumatic growth, while related to meaning-making, refers more specifically to positive psychological transformation that may emerge after trauma, such as increased personal strength, changed life priorities, or deeper self-understanding. Accordingly, this study conceptualizes coping and meaning-making as mechanisms operating within the broader resilience process, while well-being represents one important outcome associated with recovery and adaptation after IPV.

### Coping as a mechanism of resilience

Coping represents one of the central processes through which resilience is enacted. Lazarus and Folkman (1984) define coping as the cognitive and behavioral efforts individuals employ to manage demands perceived as stressful or exceeding their available resources. Coping is a dynamic process of continuous adjustment that enables individuals to adapt to adverse life experiences, including violence.

Coping strategies are commonly categorized as problem-focused or emotion-focused. Problem-focused coping involves efforts to address the source of stress or modify one’s relationship with it (Carroll, 2020). Examples include seeking information, planning, and problem-solving (Hidayat and Toybah, 2025). Such strategies are often associated with improved psychological outcomes when individuals perceive some degree of control over their circumstances (Wang et al., 2023). Emotion-focused coping, by contrast, aims to regulate emotional responses to stress, particularly when stressors are seen as uncontrollable. Strategies such as seeking social support, cognitive reappraisal, and emotional expression may help individuals manage distress and maintain psychological functioning (Hidayat and Toybah, 2025).

Individuals rarely rely exclusively on one coping style. Instead, they employ combinations of strategies that evolve over time depending on situational demands, personal resources, and cultural contexts (Lazarus, 1993). For survivors of IPV, coping strategies may include seeking help from formal or informal support networks, developing safety strategies, reframing experiences, or drawing on religious and cultural resources. These coping processes represent pathways through which resilience develops after violence (Winfield et al., 2024).

In many cultural contexts, coping processes are also shaped by socially constructed gender roles and spiritual belief systems. Expectations surrounding women’s roles as wives, mothers, and caregivers may influence how women interpret violence, tolerate abusive relationships, or seek help. At the same time, spirituality and religious beliefs can function as coping resources by providing emotional comfort, hope, meaning, and a sense of endurance during periods of adversity. Research has shown that survivors may draw upon prayer, faith, and spiritual reflection to regulate emotional distress and sustain resilience following traumatic experiences (Krok, 2015). However, these same cultural and religious frameworks may sometimes encourage women to remain silent or preserve family unity despite violence. Examining the complex role of gender roles and spirituality is therefore important for understanding women’s coping and recovery processes following IPV.

### Well-being as an outcome of resilience

Within the recovery process following IPV, restoration of well-being represents an important outcome associated with resilience and adaptation. Well-being is a concept encompassing subjective experiences and objective life conditions, including happiness, life satisfaction, and a sense of meaningful functioning (Diener, 2021). Research commonly distinguishes between subjective well-being, psychological well-being, and social well-being. Subjective well-being refers to individuals’ evaluations of their own lives, including emotional experiences and life satisfaction (Angner, 2010; Diener et al., 2018). Psychological well-being emphasizes optimal psychological functioning beyond the absence of mental illness. Ryff’s (1989) model conceptualizes psychological well-being through dimensions such as self-acceptance, personal growth, autonomy, purpose in life, environmental mastery, and positive relationships with others. Social well-being in turn reflects individuals’ integration into and contribution to their communities and social environments (Keyes, 1998).

IPV often undermines multiple dimensions of well-being and affects emotional stability, self-worth, social relationships, and economic security (Cations et al., 2021). Within a resilience framework, recovery from violence involves gradual processes through which survivors rebuild psychological functioning, regain autonomy, and re-establish meaningful social connections.

### Meaning-making and post-traumatic growth

Meaning-making represents another key mechanism through which resilience unfolds after traumatic experiences. Meaning in life refers to the sense that one’s life has coherence, purpose, and significance (Martela and Steger, 2016). According to Baumeister (1991), meaning provides individuals with a framework for interpreting life events, maintaining a sense of continuity, and sustaining self-worth. Core elements of meaning include comprehension (making sense of experiences), purpose (having goals and direction), and significance (feeling that life matters) (George and Park, 2016).

Traumatic experiences such as IPV can disrupt previously held beliefs about the world, relationships, and personal identity (Samios et al., 2020). Survivors may experience confusion, loss of purpose, or diminished self-worth following abuse. However, the process of reconstructing meaning can also support recovery and resilience. Through narrative reconstruction, reflection, and engagement with supportive relationships, survivors may reinterpret their experiences, develop new life goals, and redefine their identities beyond victimization.

Within resilience frameworks, meaning-making is understood as a process through which individuals interpret adversity, reconstruct identity, and restore coherence and purpose following traumatic experiences. In some cases, these processes may contribute to post-traumatic growth, which refers to positive psychological changes emerging after trauma, such as increased personal strength, altered priorities, and enhanced appreciation of life (Bryngeirsdottir and Halldorsdottir, 2022). Examining how survivors reconstruct meaning after IPV can provide insight into the processes that facilitate long-term recovery and well-being.

## Research gap, significance, and questions

A substantial body of research has examined coping processes, resilience, and post-traumatic growth among survivors of IPV (Hamby et al., 2018; Tsirigotis and Łuczak, 2018; Samios et al., 2020). Such studies have documented the complex ways women interpret abuse, mobilize coping strategies, and reconstruct their lives after leaving violent relationships (e.g., Stubbs and Szoeke, 2022). However, scholars have also emphasized that survivors’ coping and meaning-making processes are shaped by specific sociocultural contexts (Krok, 2015). There remains a need for qualitative research examining how women in different cultural settings experience recovery and reconstruct well-being and meaning after IPV. This study contributes to this literature by examining the lived experiences of women in Türkiye and focusing on how coping, well-being, and meaning-making processes unfold following experiences of violence.

This research is significant for exploring the sociodemographics of women experiencing IPV, its effects, and the extent of abuse. It further investigates their resilience, including coping, well-being, and meaning-making in relation to factors like age, marital status, and education. Broadly, the study aims to increase awareness of women’s value and capabilities, contributing to women’s rights and violence awareness for protection and empowerment. Given the limited research on IPV in Türkiye due to data collection challenges, this study provides insights into survivors’ difficulties, supports effective biopsychosocial services, and aids prevention. Finally, considering the scarcity of research on disadvantaged individuals and the topic’s sensitivity, this direct data collection from survivors is a valuable contribution to the field. To achieve these aims, this research seeks to answer the following questions:

1. What are the perceptions of women who experienced IPV regarding IPV?
2. What are the reasons for IPV against women?
3. What are the types of IPV experienced by women?
4. What are the strategies of women in coping after experiencing IPV?
5. What strategies do women use to make life meaningful after experiencing IPV?
6. What strategies do women use to maintain well-being after experiencing IPV?

## Method

### Research design

The research employs a qualitative research design. Qualitative research seeks to explore and understand the meaning individuals ascribe to social or human problems. It is considered a more suitable approach than quantitative methods for understanding the complexities of human experiences, particularly when studying complex phenomena such as coping, well-being, and meaning-making in the context of IPV (Yin, 2015). This approach is also appropriate because it allows for in-depth exploration of the experiences of women who have experienced IPV, focusing on their coping strategies, their journey toward well-being, and how they reconstruct meaning in their lives following abuse. This approach facilitates the examination of the underlying events and experiences that shape these women’s perceptions.

### Participants and sampling

The participants in this study comprised 16 women who had experienced IPV, with ages varying from 27 to 51 years. Five participants were married, nine were divorced, and two were single. The educational backgrounds of the women included four primary school graduates, eight high school graduates, and four university graduates. Professionally, 12 participants engaged in diverse occupations such as homemaking, civil service, manual labor, and trade. The age at which the participants first married ranged from 17 to 28 years.

The study used a combination of purposive and snowball sampling to recruit women survivors of IPV living in three cities in the Southeastern Region of Türkiye. This region is characterized by relatively traditional and collectivist social structures where gender roles, family expectations, and social stigma surrounding divorce and separation may influence women’s experiences of IPV and help-seeking processes.

Purposive sampling was initially used to recruit adult women who had experienced IPV and were connected to women’s shelters, social support services, or NGOs supporting survivors of violence. Eligibility criteria included being 18 years or older, having experienced violence within a marital or intimate partner relationship, and having ended or distanced themselves from the abusive relationship at the time of the interview to ensure participant safety and allow reflective accounts. These criteria were shared with partner organizations prior to recruitment, and service providers first informed eligible women about the study and asked whether they consented to be contacted by the researcher. Women who agreed were then approached directly, provided with full study information, and invited to participate voluntarily. In addition, snowball sampling was employed whereby initial participants referred other women with similar IPV experiences who met the inclusion criteria, allowing recruitment to proceed iteratively until the desired sample size was reached.

The determination of the final sample size was guided by the principle of data saturation, referring to the point at which additional data no longer generated substantially new insights, themes, or conceptual categories relevant to the research questions (Saunders et al., 2018). Recruitment and preliminary analysis occurred concurrently, which allowed the researchers to monitor the emergence and repetition of themes throughout the data collection process. After interviews with 16 participants, participants’ narratives began to demonstrate considerable overlap, and no substantially new themes related to coping, meaning-making, or well-being emerged. At this stage, the researchers concluded that thematic saturation had been achieved, and therefore additional participant recruitment was not pursued.

While the study included 16 unique participants, a total of 20 interviews were conducted. Follow-up interviews were carried out with three participants to deepen understanding of particularly complex experiences, clarify earlier statements, and further explore emerging themes identified during preliminary analysis. Specifically, two participants were interviewed twice, and one participant was interviewed three times. These repeat interviews enhanced the depth and richness of the dataset rather than expanding the sample size.

### Data collection tool and procedure

This study employed semi-structured interviews to explore participants’ experiences and perceptions. A set of open-ended questions was prepared, but the semi-structured format allowed flexibility for probing, follow-up questions, and exploration of unanticipated topics. The interview questions aimed to elicit detailed responses about participants’ experiences with IPV, coping strategies, their perceptions of well-being, and how they reconstructed meaning in their lives post-violence. Examples of key interview questions included the following: “What were the main ways you tried to cope with the violence while it was happening?” “What gives your life meaning and purpose today?” and “What steps did you take to begin rebuilding your life after the violence ended?” The semi-structured format also enabled the use of probing techniques to encourage deeper reflection and clarification. Probing questions included prompts such as “Could you explain that further?” “How did that experience affect you emotionally?” “What happened after that?” and “Can you give an example of what you mean?” These probes helped generate richer and detailed narratives while allowing participants flexibility in expressing their experiences.

A pilot interview was conducted to refine and enhance the clarity and relevance of the questions based on participant feedback. Minor adjustments were made to the wording of certain questions to ensure greater clarity and cultural appropriateness for the participant group. Specifically, some phrases were rephrased to avoid potential ambiguity or misunderstanding, and the sequencing of a few questions was altered to create a more natural and comfortable conversational flow, resulting in a schedule containing 15 questions (Supplementary Appendix 1).

Ethical permission was obtained from the researchers’ university ethics board prior to data collection. Following approval, preliminary meetings were conducted to explain the study’s purpose, ensure voluntary participation, and allow participants to reflect on the timeline of violence, its cessation, and their current circumstances. Participants selected their preferred interview settings (homes, cafés, or workplaces) to ensure comfort and safety. Before each interview, the study procedures, expected duration, interview content, and participants’ rights, including the right to decline questions, pause, or withdraw at any time, were clearly explained, and informed consent was obtained. Semi-structured interviews were then conducted in Turkish, audio-recorded with consent, and lasted between 40 min and 1.5 h, allowing for in-depth and flexible exploration of experiences. Verbatim transcriptions formed the dataset for analysis.

Throughout the study, particular attention was paid to ethical and trauma-informed procedures, given the sensitivity of IPV research. Participant safety and emotional well-being were prioritized by ensuring interviews took place only in participant-selected locations that afforded privacy and minimized risk of interruption or exposure. The researchers maintained a trauma-informed, non-judgmental stance grounded in empathy, active listening, and participant control over the pace and depth of disclosure, while avoiding pressure to provide overly detailed accounts of distressing events. When signs of emotional discomfort emerged, interviews were paused, and participants were offered the option to continue or stop. Participants were also informed about available psychological and social support services, including shelters and counseling centers, in case participation elicited later distress. To ensure confidentiality, all identifying information was removed, participants were assigned pseudonyms (P1–P16), and all recordings and transcripts were securely stored and accessible only to the research team.

### Data analysis, trustworthiness, and researchers’ positionality

The data collected through semi-structured interviews were analyzed using thematic analysis, a method well suited for examining participants’ lived experiences related to the phenomenon under study. The analysis began with the transcription of audio-recorded interviews to ensure an accurate textual record of participants’ narratives. Both researchers independently reviewed and coded portions of the interview transcripts. Initial codes were compared and discussed, and discrepancies were resolved through discussion until agreement was reached. These meaningful units were coded through a combination of deductive reasoning based on the study’s guiding questions and inductive interpretation, allowing new insights to emerge from the data. This coding process, the second phase of our thematic analysis, was followed by a third phase where codes were grouped to identify overarching themes. In the fourth phase, these themes were reviewed for recurring patterns and differences, as well as for internal consistency, distinctiveness, and relevance to the research objectives. The refined themes were then used to structure the findings, supported throughout by illustrative quotations from participants.

In addition, the researchers continuously monitored data saturation throughout data collection and preliminary analysis. Saturation was approached primarily from the perspective of meaning saturation rather than only code saturation. While recurring descriptive codes emerged relatively early in the analysis, the researchers continued data collection until no substantially new conceptual insights, dimensions, or interpretations related to women’s coping, well-being, and meaning-making processes were identified. By the later interviews, participants’ narratives repeatedly reflected similar patterns concerning the recognition of violence, economic dependence, emotional distress, religious coping, social stigma, rebuilding autonomy, and reconstructing meaning after violence. Follow-up interviews with selected participants were conducted to deepen understanding of already emerging themes and clarify complex experiences. After the sixteenth participant and twentieth interview, the researchers determined that additional interviews were no longer contributing new conceptual understandings relevant to the research questions, indicating that sufficient thematic and meaning saturation had been achieved.

Trustworthiness was ensured using Miles and Huberman’s (1994) criteria. Credibility was enhanced through prolonged data engagement, detailed thematic analysis, and verbatim quotes. Transferability was supported by rich contextual descriptions. Dependability was addressed through transparent documentation of data collection and analysis. Confirmability was achieved via researcher reflexivity and a clear audit trail.

The primary researcher’s psychology background and professional experience in women’s welfare institutions, including direct support work at a Women’s Shelter and the Violence Prevention and Monitoring Centre, informed the study’s overall orientation and sensitivity toward survivors of IPV. This professional background facilitated rapport building during interviews, as participants often perceived the researcher as someone familiar with the realities of violence and supportive services. At the same time, the researchers remained aware that prior professional experiences and assumptions regarding IPV, resilience, and women’s empowerment could influence data interpretation and interactions with participants. To reduce potential interviewer bias, reflexive discussions were conducted throughout the research process, and both researchers independently reviewed portions of the transcripts, compared interpretations, and discussed discrepancies before finalizing themes.

Given the sensitive nature of IPV research, emotional reflexivity was also considered throughout data collection and analysis. Some interviews involved emotionally intense narratives involving fear, trauma, and abuse, which occasionally generated emotional responses such as empathy and emotional discomfort for the researchers. The researchers therefore engaged in continuous self-reflection regarding how emotional reactions, personal values, and professional commitments might shape questioning, interpretation, and representation of participants’ experiences. Care was taken to avoid imposing preconceived interpretations on participants’ narratives and to prioritize survivors’ own perspectives throughout the analytical process.

The involvement of a second researcher, a professor of Educational Psychology with expertise in academia and social services, further strengthened the study’s credibility by providing an additional analytical perspective and critical reflection on emerging interpretations. This collaborative and reflexive approach contributed to a more nuanced understanding of women’s coping, recovery, and meaning-making processes following IPV.

## Findings

This section presents the findings obtained from the thematic analysis of interviews conducted with women who experienced IPV. The analysis generated seven overarching themes and related 31 sub-themes that illustrate how women interpreted violence, coped with abuse, rebuilt their well-being, and reconstructed meaning after the violence. The findings reveal a processual flow of recovery unfolding from recognizing violence through coping and resistance to rebuilding well-being and reconstructing life meaning. The thematic map is given in Table 1.

**Table 1.** Themes and sub-themes drawn from thematic analysis Table 1. long description.

Themes	Sub-themes
Recognizing and interpreting violence	Violence as a Violation of Human Rights (*n*:14); Violence as an Expression of Male Power (*n*:11); Violence as an Outburst of Anger (*n*:8)
The continuum of violent experiences	Violence in the Early Stages of Marriage (*n*: 8); Violence Across the Life Course (*n*: 7); Violence Within Family Relationships (*n*: 5); Violence During Pregnancy (*n*: 4)
Factors contributing to violence	Communication Breakdown (*n*: 8); Economic Dependence (*n*: 8); Neglect (*n*: 6); Early Marriage (*n*: 5); Silencing Through Exposure Threats (*n*: 4); Men’s Harmful Habits (*n*: 4)
Multiple forms of violence experienced	Physical Violence (*n*: 16); Psychological and Verbal Violence (*n*: 15); Financial Violence (*n*: 12); Sexual Violence (*n*: 6)
Coping and surviving violence	Reactions to Violence (*n*: 16); Emotional Expression (*n*: 13); Coping with Social Labeling (*n*: 12); Religious Coping (*n*: 5); Gender Roles and Endurance (*n*: 5)
Rebuilding life and well-being	Ending the Cycle of Violence (*n*: 16); Employment and Economic Independence (*n*: 13); Building Healthy Communication (*n*: 12); Personal Growth and Self-Development (*n*: 12)
Reconstructing meaning after violence	Rediscovering Positive Experiences (*n*: 14); Transforming Suffering into Strength (*n*: 12); Experiencing Success (*n*: 10); Positive Self-perception (*n*: 10); Motherhood as a Source of Purpose (*n*: 8)

*Note*: *n* indicates the number of participants whose narratives reflected the respective sub-theme. Because participants often described multiple experiences, a single participant could contribute to more than one sub-theme; therefore, frequencies do not sum to the total sample size.

Participants reported different pathways into marriage. Twelve described their marriages as arranged by their families, while four indicated that their marriages resulted from a mutual decision with their partners. When asked about the degree of autonomy in this process, nine participants stated that their families played the primary role in the marriage decision, whereas seven reported that they had actively participated in the decision together with their partner. The experiences of violence primarily occurred within their marriages or intimate partner relationships. Fourteen participants reported experiencing violence primarily from intimate partners. In addition, several participants described broader patterns of family-related violence, including emotional pressure, control, or abuse from in-laws and other family members that reinforced or normalized IPV.

### Recognizing and interpreting violence

Women’s experiences of IPV began with the gradual recognition and interpretation of violence within their relationships. This theme consists of three sub-themes that describe how participants’ understanding of abuse evolved over time, moving from normalization or minimization toward a clearer recognition of violence as unjust and oppressive.

#### Violence as a violation of human rights

Many participants ultimately framed violence as a fundamental violation of their dignity and basic rights. This interpretation reflects a shift from seeing abuse as a private marital issue to recognizing it as a broader injustice that denies women safety, autonomy, and equality. One participant expressed this realization clearly: *“It is not just about the bruises, it is about him taking away my right to feel safe in my own home, to be treated like a human being.”* (P3)

#### Violence as an expression of male power

As women reflected on their experiences, many interpreted violence as a manifestation of gendered power relations embedded within the broader social context. Participants described how expectations surrounding masculinity and male authority enabled and normalized abusive behaviors. One participant described this dynamic: *“My husband, because he was a man had the right to do anything. When I did it, he would say you are a woman. It is like he is everything and I am nothing.”* (P6) These interpretations demonstrate how women’s understanding of violence evolved from individual explanations toward broader awareness of structural gender inequality.

#### Violence as an outburst of anger

In earlier stages of their relationships, some women initially interpreted violent incidents as temporary expressions of anger rather than a pattern of abuse. This interpretation sometimes served as a coping mechanism that allowed them to rationalize the behavior and preserve hope for the relationship. As one participant reflected: *“At first, I thought it was just his temper. I would think, ‘Oh, he had a bad day at work’ but then it kept happening…”* (P11) Over time, however, repeated incidents gradually reshaped women’s understanding of the violence.

### The continuum of violent experiences

This theme consists of four sub-themes that describe the occurrence of abuse across different stages of life and how women’s understanding and response to IPV have evolved.

#### Violence in the early stages of marriage

For some women, violence emerged very early in the relationship, often within the first months of marriage. These early experiences disrupted expectations of marriage as a safe and supportive partnership. One participant recalled: *“It started so subtly - a harsh word here, a shove there - but it was there from the beginning, casting a shadow over what I thought would be a happy marriage.”* (P16)

#### Violence across the life course

Some participants described experiencing violence both before and after marriage, indicating a broader continuum of control and abuse within family structures. One woman explained this transition: *“I thought marriage would be my escape but it was just a different cage.”* (P13) These experiences suggest that IPV cannot always be understood in isolation from earlier experiences of gendered power within families.

#### Violence within family relationships

Several participants described violence as extending beyond the intimate partner relationship and involving broader family dynamics. In some cases, women experienced emotional pressure, control, or abuse from in-laws and other family members who supported or normalized the partner’s violent behavior. Participants also reported that family expectations regarding obedience, maintaining marriage, and protecting family reputation limited their ability to seek help or leave abusive environments. One participant explained: *“When I tried to tell my family what was happening, they told me to be patient and not shame the family. They said every marriage has problems.”* (P5) These experiences show that violence was often embedded within family systems that normalized gender inequality and discouraged women from resisting abuse.

#### Violence during pregnancy

Violence sometimes intensified during pregnancy, a period when women went through difficult transitional experiences and expected increased care and protection. Instead, some participants experienced heightened vulnerability. One participant described this painful contradiction: *“He knew I was carrying his child but that did not stop him. In fact, it seemed to make him angrier.”* (P2) These accounts highlight how violence can persist even during periods traditionally associated with care and family stability.

#### Factors contributing to violence

Participants identified several connected factors that contributed to the emergence and continuation of violence within their relationships. This theme consists of six sub-themes that describe the nature of IPV, comprising various factors contributing to violence.

#### Communication breakdown

Many women described persistent communication problems within their relationships. Attempts to express concerns or resolve conflicts were often met with hostility or dismissal. As one participant explained: *“He would not listen. He would just yell or walk away. It was like I did not have a voice.”* (P5)

#### Economic dependence

Economic vulnerability was identified as a major factor that limited women’s ability to resist or leave abusive relationships. Financial control often reinforced the power imbalance within the relationship. Participants emphasized that lacking independent income made them more vulnerable and constrained their options. One participant said: *“I used to work before we got married but he gradually made me resign from my work. I was totally dependent on him economically and that marked the beginning of his violence behavior.”*

#### Neglect

Participants also described emotional neglect as a harmful dimension of their relationships. Being ignored or treated as insignificant gradually eroded their sense of self-worth.

One participant expressed this experience: *“It was not just the hitting. It was the way he ignored me, like I did not matter. That kind of neglect chips away at you. Neglect was always there especially after we got married.”* (P1)

#### Early marriage

Participants who married at a young age described feeling particularly powerless within their relationships due to limited knowledge, resources, and social support. One woman explained: *“I was just a girl when I got married. I did not know anything about my rights or what a healthy relationship looked like. I was trapped.”* (P8)

#### Silencing through exposure to threats

Some participants reported that their partners used threats of exposing private information or family secrets as a means of control. These threats created fear and prevented women from seeking support. As one woman described, *“He would say, ‘If you tell anyone what is happening, I will tell everyone your secrets.’ It kept me silent for so long.”* (P15)

#### Men’s harmful habits

Substance abuse and other destructive behaviors were also described as contributing factors to violence. One participant stated: *“His drinking habit changed him. He became someone I did not recognize, and that is when the violence started.”* (P4)

### Multiple forms of violence experienced

The analysis of participants’ narratives generated four sub-themes under this overarching theme, reflecting the complex nature of IPV that involves multiple forms of abuse.

#### Physical violence

Women reported direct physical assaults such as hitting and kicking. In some cases, violence was perpetrated not only by spouses but also by other family members. One participant said: *“It was not just my husband. His mother also hit me.”* (P14)

#### Psychological and verbal violence

Psychological abuse, including insults, humiliation, and threats, was commonly described and often had long-lasting emotional consequences. For example, a participant stated, *“The words hurt just as much as the fists. He would call me names and tell me I was worthless… It wears you down.”* (P7)

#### Financial violence

Many participants experienced financial control that limited their independence and reinforced dependency. One participant stated: *“I was not allowed to work. I had to ask him for every penny. It made me feel like a prisoner.”* (P9) These experiences made men feel more control over women and have the right to exercise excessive financial authority at home.

#### Sexual violence

Participants also described sexual coercion and violations of bodily autonomy within marriage. One participant reported: *“It was not just about sex. It was about him having complete control over my body, even when I said no.”* (P10)

Analysis of participants’ narratives in relation to their sociodemographic backgrounds revealed important patterns in how IPV was experienced and interpreted. Women who entered marriage at an early age and those with lower educational attainment more frequently described limited awareness of rights, restricted autonomy in decision-making, and greater normalization of violence within marriage. In contrast, participants with higher educational levels more often framed violence in terms of rights violations and expressed earlier awareness of alternative life options. Employment status also shaped experiences, as women without independent income reported stronger financial dependence and reduced capacity to leave abusive relationships. Across marital status, divorced participants more frequently described a gradual process of resistance and eventual separation, whereas currently married participants tended to emphasize ongoing negotiation, endurance, or constrained coping strategies.

### Coping and surviving violence

Despite the severity of violence, women demonstrated diverse strategies for coping with and surviving their experiences. This theme consists of five sub-themes that describe these strategies, reflecting various forms of resilience developed in response to adversity.

#### Reactions to violence

When confronted with violence, women described a range of responses, including silence, resistance, leaving their homes, or seeking help. Some participants stated: *“I used to keep quiet and try to ignore what was happening”* (P10) and *“I had to run away from home to protect myself.”* (P14) These reactions were often shaped by safety considerations and resources.

#### Emotional expression

Expressing emotions was another way women coped with the psychological burden of violence. For example, a participant said: *“Crying was my release. It was the only way I could express the pain.”* (P12)

#### Coping with social labeling

Women also described the social stigma associated with divorce or leaving abusive relationships. One participant illustrated: *“The word ‘divorced’ felt like a brand. People treated me differently, like I was damaged.”* (P4) Experiencing these social labels became an additional challenge during their recovery process.

#### Religious coping

For several participants, faith and spirituality served as important sources of comfort, emotional regulation, and strength during and after experiences of violence.

A participant reported: *“Turning to God gave me the strength to keep going. Prayer was my refuge.”* (P7) Participants described prayer, spiritual reflection, and trust in God as helping them manage fear, loneliness, and emotional exhaustion while sustaining hope for a safer future.

#### Gender roles and endurance

Participants’ narratives reflected the influence of socially constructed gender roles on how they interpreted and coped with violence. Several women described feeling pressure to endure abuse because they had been taught that women should be patient, preserve family unity, and prioritize caregiving responsibilities over their own well-being. In many cases, these expectations delayed help-seeking and contributed to the normalization of suffering within marriage. One participant explained: “*I kept telling myself that a mother should keep the family together no matter what. I thought enduring everything was part of being a good wife and mother.”* (P9) Motherhood was also a particularly important dimension shaping women’s coping processes. While some participants initially viewed motherhood as a reason to remain in violent relationships, others gradually reinterpreted their caregiving role in more empowering ways and began to prioritize safety and well-being for themselves and their children. As one participant stated: *“One day I realized that protecting my children also meant protecting myself. I did not want them to grow up believing violence was normal.”* (P2)

Coping strategies varied across participants’ sociodemographic positions. Women with stable employment and higher education were more likely to report active coping strategies such as seeking formal support services, legal assistance, or initiating separation processes. In contrast, participants with lower socioeconomic status more frequently relied on emotion-focused coping strategies such as endurance, emotional suppression, or religious coping. Mothers, particularly those with dependent children, described coping strategies shaped by caregiving responsibilities, often prioritizing children’s safety over their own well-being. Marital status further shaped coping trajectories, as divorced participants reflected retrospectively on coping transitions that included escalation from silence to resistance, while married participants often remained in earlier phases of coping characterized by adaptation and constraint.

### Rebuilding life and well-being

Participants described working toward rebuilding their lives after IPV. These efforts, reported under four sub-themes, reflected attempts to regain autonomy and restore well-being.

#### Ending the cycle of violence

Leaving abusive relationships was rarely a single and abrupt decision but rather a gradual process involving reflection and planning. One participant illustrated this process: *“Leaving was not a single moment, it was a long journey of weighing my options and finding the strength to choose myself.”* (P4) Often associated with their resilient stance, women decided to end violence by utilizing various resources such as personal decisiveness, self-belief, and social support.

#### Employment and economic Independence

Employment played a crucial role in restoring independence and self-worth. A participant said: *“Having my own job gave me a sense of worth and the ability to provide for myself. It was a turning point.”* (P12)

#### Building healthy communication

After experiencing abuse, women often prioritize building healthy communication patterns in their new relationships, setting boundaries, and asserting their needs to prevent future abuse and create healthier connections. This focus on healthy communication is a key aspect of rebuilding trust and safety. As one participant articulated: *“I learned that I have a right to speak up, to say no and to expect respect in my relationships.”* (P1)

#### Personal growth and self-development

Women actively worked on their personal growth and development as part of their journey toward well-being, focusing on self-improvement and empowerment. Self-improvement is a way to reclaim their lives and build a stronger sense of self. For example, two participants said: *“I started taking classes, reading books and focusing on myself,”* one participant shared. *“I wanted to become the best version of myself, not the broken person I once was.”* (P16) These efforts toward well-being demonstrate women’s active role in rebuilding their lives.

### Reconstructing meaning after violence

Beyond rebuilding practical aspects of life, participants described deeper processes of meaning-making that allowed them to reinterpret their experiences and move forward. Meaning-making involved several tools, means, and forms that are reported under five sub-themes.

#### Rediscovering positive experiences

Rediscovering positive experiences and emotions is a part of finding meaning and joy in life after abuse. This reconnection with positive experiences is crucial for healing. One participant said, *“After so much darkness*, *I focused on finding the light again - in nature, in art, in the kindness of others.”* (P6) These experiences illustrate the importance of recovery from a cycle of negative experiences and discovering favorable life experiences and relationships.

#### Transforming suffering into strength

Women can transform their experiences of violence into a source of strength and empowerment and view their survival as a testament to their resilience and agency. A participant illustrated: *“I used to see myself as a victim but now I see myself as a survivor. I am stronger because of what I went through.”* (P8) Such experiences illustrate how resilience can help women draw conclusions from their experiences and apply new ways of constructing life.

#### Experiencing success

Achieving goals and experiencing success in different areas of life, such as education, career, or personal pursuits, can contribute to a woman’s sense of self-worth and meaning. *“Setting goals and achieving them, no matter how small, gave me a sense of accomplishment and showed me what I was capable of,”* a participant stated. (P13)

#### Positive self-perception

Developing a positive self-image and self-acceptance is crucial for healing and building a meaningful life after abuse. *“Learning to love myself again, to believe in myself – that was the hardest part, but it was also the most important,”* one woman shared. (P10) These methods of creating meaning show the resilience of women in reconstructing their lives after violence.

#### Motherhood as a source of purpose

Motherhood is a source of meaning and motivation for many women. It gives them strength, purpose, and a reason to rebuild their lives. For example, a participant said: *“My children gave me a reason to keep going, to fight for a better future. They were my strength.”* (P2) Embarking on this source of power, women could search for ways of making meaning.

Overall, although thematic patterns were shared across participants, the expression and intensity of coping, resistance, and meaning-making processes were influenced by variations in education, employment status, marital status, motherhood, and socioeconomic positioning. These differences suggest that resilience processes are not uniform but are shaped by structural and life-course inequalities.

## Discussion

This research explored the experiences of women who have confronted and overcome IPV. The findings reveal an interplay of factors shaping women’s perceptions of violence, their resilience in coping with abuse, and their pursuit of a meaningful life after violence. The findings indicate that women’s coping and recovery processes were shaped by intersecting sociodemographic factors, including education, employment, marital status, motherhood, and socioeconomic positioning. Higher educational attainment appeared to facilitate earlier recognition of violence and access to support resources, while economic independence emerged as a critical enabling factor for leaving abusive relationships. Conversely, lower socioeconomic positioning was associated with increased financial dependence and reliance on endurance-based coping strategies. These findings align with intersectional perspectives on IPV, which emphasize that women’s experiences of violence are not uniform but are structured by overlapping social inequalities that shape both vulnerability and resilience (Richardson and Taylor, 2009). Recognizing these differences is essential for developing interventions that address the specific needs of diverse groups of survivors.

### Resilience after intimate partner violence

The experiences described by the participants illustrate that women’s responses to IPV are not limited to victimization but also involve processes of adaptation, coping, and rebuilding their lives. These processes can be understood through the concept of resilience, which refers to the dynamic capacity of individuals to navigate adversity and gradually restore well-being despite exposure to stress or trauma (Masten, 2001). In the narratives shared by the participants, resilience becomes visible in the ways women reinterpret violence, develop strategies to cope with abuse, and pursue new pathways toward safety, autonomy, and personal growth.

Resilience in the context of IPV is increasingly conceptualized as a process shaped by individual resources, social relationships, and cultural environments (Anderson et al., 2012). The accounts of the women in this study reflect this multidimensional process. For instance, participants described turning to emotional expression, social support, and religious faith to endure the psychological impact of violence. Others emphasized the importance of employment, personal development, and rebuilding communication in relationships as part of their efforts to regain independence and stability. These experiences demonstrate how resilience emerges through the interaction of personal agency and available social and cultural resources.

Within this study, resilience provides a lens for interpreting the themes emerging from the data. Women’s evolving perceptions of violence reflect the process of recognizing and redefining abuse, while their coping strategies illustrate how survivors manage the immediate consequences of violence. Their efforts to rebuild well-being and create meaning in their lives demonstrate longer term processes of recovery, identity reconstruction, and personal growth. Together, these dimensions illustrate how resilience unfolds as a gradual process through which women move from surviving violence toward rebuilding autonomy, purpose, and well-being.

The findings further extend resilience-oriented understandings of IPV by suggesting that resilience among women survivors in southeastern Türkiye is not solely an individual psychological capacity but a culturally negotiated, relational, and structurally constrained process. Women’s efforts to cope with violence and reconstruct their lives were shaped by patriarchal family systems, collectivist expectations surrounding marriage and motherhood, economic dependency, and moral pressures related to family unity and social reputation. In this context, resilience emerged not simply through individual empowerment but through women’s ongoing negotiation with restrictive social norms, family obligations, and gendered expectations. This finding contributes to resilience literature by demonstrating that resilience in collectivist and patriarchal contexts may develop differently from more individualistic Western conceptualizations that primarily emphasize autonomy, self-efficacy, and personal independence.

### Recognizing violence as a turning point in the resilience process

The findings suggest that recovery from IPV begins with a cognitive shift in how women interpret violence. Many participants initially framed abusive acts as isolated outbursts of anger and reflected attempts to normalize or minimize the situation. Such interpretations are consistent with psychological processes such as cognitive dissonance, where individuals attempt to reconcile conflicting experiences within intimate relationships (Arman et al., 2025). Over time, however, participants’ interpretations evolved. Violence increasingly came to be understood as a violation of fundamental human rights and as a manifestation of gendered power relations.

This shift in perception represents an important turning point in the resilience process. Resilience research emphasizes that adaptive responses to adversity often begin with the recognition of structural constraints shaping individual experiences (Forsdike and O’Sullivan, 2024). In the case of IPV, recognizing violence as a form of systemic gender inequality rather than a personal failure allows survivors to reinterpret their experiences and begin reclaiming agency (Ceylan-Batur et al., 2023). The participants’ narratives, therefore, reflect a transition from internalized explanations of violence to structural awareness, aligning with feminist scholarship that conceptualizes IPV as embedded within patriarchal social norms (Grose et al., 2021).

The normalization of violence described by many participants also appears closely connected to sociocultural processes operating within the regional context of Türkiye. Several women described growing up in environments where male authority, women’s endurance, and silence surrounding family problems were normalized within both natal and marital families. In these contexts, violence was not always immediately recognized as abuse but was often interpreted as a regrettable yet ordinary aspect of marriage. This finding suggests that the normalization of IPV cannot be understood solely through individual psychological mechanisms such as denial or minimization. Rather, normalization appears socially reproduced through intergenerational gender norms, collectivist pressures, and cultural expectations emphasizing patience, sacrifice, and preservation of family unity. Such findings deepen existing literature by illustrating how patriarchal socialization shapes not only women’s vulnerability to violence but also the ways violence is initially interpreted and tolerated.

The findings further suggest that IPV should be understood not solely as an individual or couple-level problem but also as a phenomenon shaped by broader family systems and intergenerational gender norms. Several participants described how family members, including parents and in-laws, normalized violence, discouraged separation, or prioritized family reputation over women’s safety. Such responses may contribute to the silencing of abuse and reinforce women’s dependence within violent relationships. Prior research similarly indicates that family environments can reproduce patriarchal norms by legitimizing male authority and encouraging women’s endurance of abuse for the preservation of family unity (True, 2021). In this study, some women also described experiences of violence or control before marriage, suggesting continuity between violence experienced within natal families and later intimate relationships. These patterns highlight the importance of family-centered prevention and intervention approaches that address intergenerational attitudes toward gender, power, and violence.

The findings indicate that family systems within this sociocultural context simultaneously functioned as both sources of support and mechanisms reinforcing women’s endurance of violence. While some participants eventually received emotional or practical assistance from relatives, many others described being encouraged to remain patient, avoid divorce, or protect family reputation despite ongoing abuse (Dolkun et al., 2025). This contradiction highlights a tension within women’s resilience processes: resilience often emerged not because women were surrounded by consistently supportive environments but despite social systems that normalized violence and constrained women’s autonomy. This finding differs from some resilience literature emphasizing the uniformly protective role of social support and instead suggests that support networks in collectivist contexts may simultaneously empower and silence survivors.

The findings also highlight that women’s experiences of violence often unfold across the life course, including exposure to violence before marriage and continuing within intimate partnerships. This highlights the pervasive nature of violence across the lifespan and necessitates a comprehensive approach. Early violence in intimate relationships can severely impact trust and create lasting insecurity (Stöckl and Sorenson, 2024). Prior research links early exposure to violence with long-term issues in psychological development and relationships (Samios et al., 2020). Adverse childhood experiences studies demonstrate a correlation between childhood trauma, including witnessing IPV, and increased risks of adult IPV and mental health issues (Stöckl and Sorenson, 2024). Such patterns illustrate how resilience is not a single event but a process that develops within long-term structural constraints. Understanding IPV through this lens emphasizes the importance of early prevention and highlights how resilience emerges despite repeated exposure to violence.

The findings indicate that IPV in the participants’ experiences emerges from a combination of relational, economic, and social factors, including communication breakdowns between partners, women’s financial dependence, emotional neglect, threats related to revealing family secrets, early marriage, and men’s negative habits such as substance abuse. This pattern supports a multifactorial understanding of IPV that recognizes the interaction of individual, relational, and structural influences rather than attributing violence to a single cause. In particular, women’s economic dependence was a significant constraint shaping their ability to respond to abuse, consistent with social exchange theory, which suggests that individuals may remain in harmful relationships when they perceive limited alternatives or resources (Alkan et al., 2021). Financial control can therefore function as a mechanism through which abusers maintain power and restrict women’s autonomy (Ceylan-Batur et al., 2023). Communication breakdowns also appeared in participants’ accounts, reflecting findings in prior research emphasizing how unresolved conflict, lack of mutual respect, and ineffective communication may contribute to escalating tensions within intimate relationships (Cannon et al., 2021).

The participants also described experiencing multiple forms of violence, including physical, psychological, verbal, economic, and sexual abuse. These accounts reinforce existing scholarship highlighting that IPV is a multidimensional phenomenon in which different forms of violence often co-occur and interact to create a broader system of coercion and control (Mondal and Paul, 2021). In particular, the narratives illustrate the profound impact of psychological abuse, which can erode self-esteem, autonomy, and mental health even in the absence of visible physical harm (Cations et al., 2021). Research on coercive control similarly demonstrates how intimidation, isolation, and manipulation may operate as subtle but powerful mechanisms through which perpetrators maintain dominance over their partners (Alkan et al., 2022).

### Adaptive coping and survival

The diverse coping strategies employed by the participants, including emotional reactions, disclosing emotions, religious coping, and managing societal labeling, highlight the active role women take in navigating the immediate and long-term aftermath of violence. Seeking social support was a critical strategy, aligning with extensive research that emphasizes the protective role of social networks and social capital in mitigating the detrimental effects of abuse and fostering resilience (Tsirigotis and Łuczak, 2018; Johnson and Winter, 2023). Strong social connections offer vital emotional validation, practical assistance, and a sense of belonging, all of which are foundational for healing and recovery. The act of disclosing emotions, often through verbalization or even tears, as expressed by one participant, serves as a crucial step in processing trauma and breaking the isolation frequently imposed by abusive relationships. This act of sharing can be a powerful catalyst for seeking further support and initiating the healing process.

The utilization of religious coping, where faith and spirituality provide solace and strength, resonates with studies highlighting the significance of these resources for some survivors (Krok, 2015). Religion can offer a framework for understanding traumatic experiences, fostering hope, and providing a sense of community. However, support services should approach this aspect with sensitivity and recognize that religious coping can manifest in diverse ways, some of which may inadvertently reinforce passivity or acceptance of abuse. Therefore, resources should aim to empower survivors within their faith context and promote autonomy and self-determination. Furthermore, the challenge of coping with negative societal labeling, particularly the stigma associated with divorce, reveals an additional layer of adversity faced by these women. This judgment can impact self-esteem and social reintegration and thus necessitate interventions that address societal attitudes and provide support for women navigating these social stigmas.

The significance of religious coping may also reflect the sociocultural realities of southeastern Türkiye, where spiritual belief systems may constitute central sources of meaning, emotional endurance, and social identity. In contexts where discussing IPV publicly may carry stigma and where access to psychological support services may be limited, prayer and spiritual reflection may become culturally accessible mechanisms for emotional regulation and hope. At the same time, the findings reveal an important contradiction: while spirituality provided emotional comfort and resilience for many participants, certain interpretations of religious or cultural expectations surrounding patience, sacrifice, and family preservation could also contribute to prolonged endurance of abusive relationships. This demonstrates the complex and sometimes contradictory role of religion within women’s coping processes.

The findings indicate that coping and resilience were shaped by culturally embedded understandings of women’s social roles, particularly motherhood, caregiving, and expectations of female endurance. Some participants described remaining in abusive relationships because they perceived self-sacrifice, patience, and family unity as central aspects of being a “good woman” or “good mother.” These findings align with research demonstrating that gender role socialization may contribute to the normalization of women’s suffering within patriarchal contexts (True, 2021). At the same time, motherhood emerged as a transformative source of agency and resistance. For some women, concern for their children motivated help-seeking, leaving abusive environments and rebuilding safer lives. This dual role of motherhood illustrates how gendered expectations may simultaneously constrain and empower women during recovery processes.

Compared with many Western studies emphasizing individualized coping and independence-oriented recovery trajectories, the women’s experiences in this study reflected more relationally negotiated forms of resilience shaped by caregiving responsibilities, family expectations, and collective social norms. Decisions regarding leaving abusive relationships were rarely framed solely around personal fulfillment or self-actualization but were closely connected to concerns regarding children, family honor, economic survival, and social judgment. These findings suggest that resilience processes are culturally situated and that theories developed primarily within Western individualistic contexts may not fully capture the sociocultural complexity of women’s recovery experiences in more collectivist environments.

### Reclaiming autonomy and empowerment

The active steps taken by the participants to enhance their well-being, including the complex process of ending violence, pursuing employment, establishing healthy communication patterns, and engaging in self-improvement, indicate their agency and resilience in reclaiming their lives. The decision to end violence is rarely a singular event but rather a protracted and often perilous journey, fraught with internal conflict, fear, economic constraints, and concerns for the safety of children. Comprehensive support services are crucial during this critical phase, offering safety planning, legal aid, access to secure housing, and emotional support tailored to the individual’s circumstances. The pursuit of employment emerged as a significant step toward independence and empowerment, aligning with feminist economic perspectives that emphasize the link between economic self-sufficiency and improved well-being, as well as reduced vulnerability to future abuse (Dolan and Stancanelli, 2021). Access to education, vocational training, and equitable employment opportunities can empower women to leave abusive environments, establish financial security, and regain control over their lives and futures.

Participants’ efforts to establish healthy communication patterns in new relationships signify a proactive step toward preventing future abuse and fostering healthier connections. This involves developing assertiveness skills, setting clear boundaries, and cultivating mutual respect in interactions. Learning to recognize and challenge unhealthy communication dynamics is a vital aspect of rebuilding trust and safety in future relationships. Finally, the participants’ engagement in self-improvement and personal development highlights a powerful drive toward reclaiming their sense of self and building a stronger, more resilient identity post-abuse. This active pursuit of growth, whether through education, personal interests, or self-reflection, demonstrates a commitment to moving beyond the trauma and creating a fulfilling future.

### Recovery and meaning reconstruction

The diverse methods employed by the participants to reconstruct meaning in their lives after experiencing violence, including reconnecting with positive experiences, finding purpose in motherhood, reframing their struggles as empowering, achieving personal and professional success, and cultivating a positive self-perception, align with the concept of post-traumatic growth (Bryngeirsdottir and Halldorsdottir, 2022). This framework suggests that even in the aftermath of profound adversity, individuals can experience positive psychological changes such as a heightened appreciation for life, strengthened relationships, an enhanced sense of personal strength, and a deeper existential understanding. The participants’ ability to find joy in simple pleasures and reconnect with positive emotions serves as a crucial counterbalance to the negativity and trauma they endured, fostering hope and a renewed sense of possibility.

For some participants, motherhood was a powerful source of meaning and motivation, providing a strong impetus to rebuild their lives and strive for a better future for their children. While acknowledging the significance of caregiving roles for many women, it is crucial for support services to avoid essentializing women’s experiences solely through the lens of motherhood (Leanderz et al., 2025). Recognizing the diverse ways in which women find meaning is paramount, and support should be individualized and aligned with each woman’s values and aspirations. The act of reframing traumatic experiences that view survival as a testament to personal strength and resilience shows the human capacity for growth in the face of adversity. Similarly, achieving success in various domains of life contributes to a renewed sense of self-worth and agency. Finally, the conscious effort to develop a positive self-perception and cultivate self-acceptance is a fundamental aspect of healing and building a meaningful life after abuse, laying the groundwork for future well-being and fulfilling relationships.

Overall, this study contributes new knowledge to IPV and resilience scholarship by demonstrating that women’s resilience in Türkiye develops within environments characterized by intersecting structural constraints, including patriarchal gender norms, collectivist family systems, economic dependency, and sociocultural expectations surrounding endurance and family preservation. The findings highlight that resilience should not be understood as a linear or purely empowering process but rather as a dynamic negotiation involving contradictions, tensions, and culturally embedded meanings. In particular, the study contributes a culturally grounded understanding of how motherhood, spirituality, and family relationships can simultaneously function as sources of resilience and mechanisms sustaining women’s prolonged exposure to violence. These findings underscore the importance of culturally responsive intervention models that recognize the relational and sociostructural dimensions of women’s recovery following IPV.

## Limitations, recommendations, and conclusion

The findings of this research are specifically limited to the data obtained from the 16 women who participated in the interviews. As such, it is not possible to generalize these findings to represent the experiences of all women who have experienced IPV. The interpretation of the findings is primarily rooted in each participant woman’s individual structure. However, the interpretation also incorporates national and international literature to draw broader inferences within the context of international research. Additionally, because participants were recruited through shelters and NGOs, the sample may represent women who had already accessed support services. Survivors who remain in abusive relationships or who have limited access to support networks may have different experiences that are not represented in this study.

The study’s findings indicate a correlation between experiencing violence and women’s mental health, recommending increased accessibility of psychological support services, potentially integrated within existing support institutions. Recognizing the crucial role of social support, fostering robust networks through support groups and peer counseling programs is advised. Given the significant influence of having children on decisions regarding abusive relationships, interventions should specifically address the unique needs and safety concerns of mothers and their children. To address the fear and distrust preventing some women from seeking formal help, trust-building efforts are crucial, involving community outreach and sensitive, supportive service delivery. Economic dependence being a major barrier to leaving, programs enhancing women’s financial independence are strongly recommended. Finally, further research is emphasized to explore the long-term impacts of IPV and facilitate the development of more effective intervention strategies.

This research contributes to a rich understanding of the experiences of women who have faced and overcome IPV. It highlights the significance of addressing the complex interplay of factors that influence women’s coping strategies, their journey toward achieving well-being, and their process of reconstructing meaning in their lives following experiences of violence. The findings of this study not only underscore the resilience and agency demonstrated by these women but also emphasize the ongoing need for concerted efforts to support and empower them in their journey toward recovery and a life free from violence.

## Supporting information

10.1017/gmh.2026.10262.sm001 Tini and Sakiz supplementary material Tini and Sakiz supplementary material

## Data Availability

Data are available upon request from the authors.

## Supplementary material

The supplementary material for this article can be found at http://doi.org/10.1017/gmh.2026.10262.

## Long descriptions

### Table 1. Long description

This table summarizes the themes identified through thematic analysis of women’s experiences of intimate partner violence. Seven overarching themes were identified: recognizing and interpreting violence, the continuum of violent experiences, factors contributing to violence, multiple forms of violence experienced, coping and surviving violence, rebuilding life and well-being, and reconstructing meaning after violence. Participants described violence as a violation of rights and an expression of power, occurring across different stages of life and relationships. They reported physical, psychological, financial, and sexual abuse, identified personal and social factors contributing to violence, and described various coping strategies. The findings also highlight processes of recovery, empowerment, personal growth, and meaning-making following experiences of violence.

Navigate back to Table 1..
