# Supplementary material for: Resilience among women survivors of intimate partner violence: Coping, meaning-making, and well-being
Source: Glob Ment Health (Camb). 2026 Jun 25;13:e151. doi: 10.1017/gmh.2026.10262 (PMC13373261; doi:10.1017/gmh.2026.10262)
Supplement: Tini and Sakiz supplementary material [file S2054425126102623sup001.docx]

**Appendix 1.** Interview questions

1. Could you share your experiences with domestic violence, including when it began and the different forms it took?
2. How did you understand what was happening to you, and did you tell anyone about it?
3. What were the main ways you tried to cope with the violence while it was happening?
4. Could you describe the key factors or turning points that led to the violence ending in your life?
5. What kind of support did you seek or receive when trying to end the violence?
6. What were the biggest challenges you faced in ending the violence?
7. What steps did you take to begin rebuilding your life after the violence ended?
8. How did things like work, new relationships, or personal development contribute to your well-being?
9. What does “well-being” mean to you now?
10. What are the most important things that contribute to your current sense of well-being?
11. How has your experience with violence changed your perspective on life?
12. What gives your life meaning and purpose today?
13. Looking back, what was most crucial in helping you end the violence and rebuild your life?
14. What are your hopes and goals for the future?
15. Is there anything else you would like to share about your experiences?
