# Supplementary material for: Resilience among women survivors of intimate partner violence: Coping, meaning-making, and well-being
Source: Glob Ment Health (Camb). 2026 Jun 25;13:e151. doi: 10.1017/gmh.2026.10262 (PMC13373261; doi:10.1017/gmh.2026.10262)
Supplement: Peer review history

# Reviewer report: Review: Resilience among women survivors of intimate partner violence: Coping, meaning-making, and well-being — R0/PR2

**DOI:** 10.1017/gmh.2026.10262.pr2

1. Please clarify why used only 16 participants instead of 20. Saturated or not?

2. Add some aspects of family issue or the influences of family members violence situation.

3. Add women' s roles or spiritual beliefs and their religious in terms of coping with violence.

# Reviewer report: Review: Resilience among women survivors of intimate partner violence: Coping, meaning-making, and well-being — R0/PR3

**DOI:** 10.1017/gmh.2026.10262.pr3

**Authors:** Asiya Muhammad

**Affiliations:** Clinical Services Department, Federal Neuro-Psychiatric Hospital Kaduna, Nigeria

Title of the manuscript: Resilience Among Women Survivors of Intimate Partner Violence: Coping, Meaning Making and Well-Being

Dear researcher(s), you are addressing an important and meaningful gap. Your paper is well-written and it has some important results, and if you edit your paper it can be much more effective. Here are some humble suggestions to improve the paper, I would do the following to strengthen the paper.

Main points:

1. Title: “Resilience Among Women Survivors of Intimate Partner Violence: Coping, Meaning Making and Well-Being”: Its relevant.

2. Abstract and keywords: clear and comprehensive.

3. Overall language: The language is fairly written.

a. You could improve on the writing.

- Examples include:

• missing commas.

• repetitive sentence structures.

• inconsistent tense usage.

• Example “IPV often undermine dimenens of well-being…”

• Should read: “IPV often undermine dimenens of well-being…”

Professional language editing is recommended.

b. However, you have to re-write/summarize some parts eg findings, discussion etc

4. Length of paragraph : Good

You can check the paper and make sure every paragraph is not more than 5 sentences. The best is to stick with 3 to 5 sentences.

5. Introduction: Good

6. Thoroughness of the literature review: Fair

a. Conceptualization: Confusion between coping, resilience, wellbeing, and meaning making. The manuscript treats, coping, resilience, well-being, meaning making, post-traumatic growth as overlapping constructs without clearly distinguishing them conceptually. For example:

- resilience is described as both a process and an outcome,

- well-being appears both as a component and endpoint of resilience,

- meaning making overlaps with post-traumatic growth.

This creates conceptual redundancy. Recommendation

- clearer operational distinctions,

- conceptual model,

- explicit explanation of relationships among constructs.

7. Research design: is good and was indicated.

8. Clearly providing research questions and/or purpose: is good, as it has been indicated in the aims, but some journals may require hypothoses.

9. Choice of research method: Is good. But can you improve on the following:

a. Interview Guide: You mention 15 interview questions but do not provide:

i. examples of key questions.

ii. probing techniques.

b. Reflexive Depth

Although positionality is briefly acknowledged, reflexivity is superficial.

You did not discuss:

i. how their professional roles influenced interviews

ii. possible interviewer bias.

iii. emotional responses during interviews.

Given the sensitivity of IPV research, reflexivity is crucial.

c. Data Saturation poorly explained

The manuscript states saturation was achieved but does not explain:

i. how saturation was determined

ii. which themes became repetitive

iii. whether code saturation or meaning saturation was used.

10. Ethical Procedures Need Expansion: Fairly written. The ethics section is minimal. Important issues are insufficiently addressed:

i. participant safety protocols.

ii. trauma-informed interviewing.

iii. psychological support after interviews.

iv. confidentiality safeguards.

This is especially important in IPV research.

11. Appropriateness of procedures chosen for data collection and analysis: Good. Cultural Context Is Underdeveloped. Although the study is situated in southeastern Türkiye, the sociocultural context is insufficiently analyzed. The manuscript briefly mentions: patriarchy, gender inequality, arranged marriage, stigma. However, it did not deeply examine: regional conservatism, ethnic/cultural influences, religion-politics intersections, family honor dynamics, collectivist pressures, rural versus urban differences. This is a missed opportunity.

12. Relevance of data obtained in view of the purpose of the research: Fair. This can be improved by analyzing differences by: Education, employment, marital status, age, motherhood, socioeconomic class. Moreso the sociodemographic variables were part of your research question.

13. Discussion of the results and their significance: Fairly written.

You can improve this section:

A) a. What new theoretical understanding emerges,

b. How Turkish sociocultural dynamics uniquely shape resilience.

c. Explain what is uniquely Turkish or region-specific.

B) Reduce some repetitions of findings in the discussion.

C) What Stronger Analytical Discussion Usually Includes:

A stronger discussion would:

• compare findings with conflicting studies,

• explain surprising results,

• discuss cultural/theoretical implications,

• identify tensions or contradictions,

• explain what new knowledge the study contributes.

For example:

• Why was religion especially important in this region?

• Why did women normalize violence initially?

• How does Turkish patriarchal culture shape resilience differently from Western findings?

• Did resilience emerge despite support systems rather than because of them?

14. Soundness of conclusions in relation to data presented: Good.

15. Limitation: well-written.

16. Implication/Recommendation: Well written.

17. Format of referencing :You have to follow what the journal indicates.

18. Figure/tables: The table 1 absent.

19. In-text reference: well written

20. References: Good

- Please use the following link to include all available doi numbers https://doi.crossref.org/simpleTextQuery simply include your reference one or more than one at a time and submit it. Then you should get all doi numbers if a manuscript has it.

I have enjoyed reading your paper and learned a lot- thanks for your contribution to social sciences. You are addressing an important and meaningful gap. Your paper has some important results, and if you edit your paper based on all or some of the humble suggestions above, it can be much more effective. I am looking forward to seeing the paper published after making the suggestions made.

Muhammad Asiya

Consultant Psychiatrist

Federal Neuro-psychiatric Hospital Kaduna,

Nigeria

Dear Authors

Here are some detailed comments of your manuscript “Resilience Among Women Survivors of Intimate Partner Violence: Coping, Meaning Making and Well-Being”

1. Page 10 -11, line 31, section: Participants and Sampling.

A. Re-write and summarize the whole section.

B. Page 10, line 35 – 47 & page 11, line 1 -15, starting from “Their ages……………….” And ends with “family members in addition to IPV.” Should be in findings.

C. It should contain what you wrote on page 11, line 17-36 e.g – i. The participants (IPV) ii. Region iii. iv. Sampling technique v. How the participants were selected.

D. Page 11, line 31-36 and line 38-45 should be on same paragraph.

E. Page 11, Line 45-50 can deleted as its not necessary.

2. Page 12, Line 8, Section: Data Collection Tool and Procedure

F. Page 12, Line 36-38 i.e “Participants were initially contacted via women’s shelters and NGOs, who identified eligible survivors.” Because its repetition

3. Findings

G. Bring the earlier findings here as you were advised on.

H. The subheading under findings should be clear eg Themes and its numbers, Factors contributing to IPV and its no., Experiencing Multiple Forms of Violence (change to Multiple Forms of Violence experienced), Coping and Surviving Violence and their no., Reactions Developed Against Violence should be clear subheadings

I. State the number of themes found in the study

4. Discussion

J. You need to discuss this objective “This research is significant for exploring the sociodemographics of women experiencing IPV, its effects and the extent of abuse.”

K. You need to discuss this objective “It further investigates their resilience including coping, well-being, and meaning making in relation to factors like age, marital status and education.”

# Editor report: Recommendation: Resilience among women survivors of intimate partner violence: Coping, meaning-making, and well-being — R0/PR4

**DOI:** 10.1017/gmh.2026.10262.pr4

**Affiliations:** Psychology, UNISA, South Africa

Dear Dr. Sakiz, 

Manuscript ID GMH-2026-0132 entitled “Resilience Among Women Survivors of Intimate Partner Violence: Coping, Meaning Making and Well-Being,” which you submitted to Cambridge Prisms: Global Mental Health, has been reviewed. The comments of the reviewer(s) and editor(s) are included at the bottom of this letter.

I invite you to revise the manuscript in accordance with the reviewers' and the editor’s comments below and submit a revised version.

When submitting your revised manuscript, you will respond to the comments made by the reviewer(s). In order to expedite the processing of the revised manuscript, please be as specific as possible in your response to the reviewer(s).

Once again, thank you for submitting your manuscript to Cambridge Prisms: Global Mental Health, and I look forward to receiving your revision. 

Sincerely, 

Dr Limkile Mpofu

Handling Editor, Cambridge Prisms: Global Mental Health

Reviewer Comments

Reviewer 1 Comments to the Author

1. Please clarify why you used only 16 participants instead of 20. Saturated or not?

2. Add some aspects of family issues or the influences of family members' violent situations.

3. Add women' s roles or spiritual beliefs and their religious beliefs in terms of coping with violence.

Reviewer 2 Comments to the Author

Dear researcher(s), you are addressing an important and meaningful gap. Your paper is well-written, and it has some important results; if you edit your paper, it can be much more effective. Here are some humble suggestions to improve the paper. I would do the following to strengthen the paper.

Main points:

1. Title: “Resilience Among Women Survivors of Intimate Partner Violence: Coping, Meaning Making and Well-Being”: It’s relevant.

2. Abstract and keywords: clear and comprehensive.

3. Overall language: The language is fairly written.

a. You could improve on the writing.

- Examples include:

• missing commas.

• repetitive sentence structures.

• inconsistent tense usage.

• Example “IPV often undermine dimenens of well-being…”

• Should read: “IPV often undermine dimenens of well-being…”

Professional language editing is recommended.

b. However, you have to re-write/summarize some parts, e.g., findings, discussion etc

4. Length of paragraph: Good

You can check the paper and make sure every paragraph is not more than 5 sentences. The best is to stick with 3 to 5 sentences.

5. Introduction: Good

6. Thoroughness of the literature review: Fair

a. Conceptualization: Confusion between coping, resilience, well-being, and meaning making. The manuscript treats coping, resilience, well-being, meaning making, and post-traumatic growth as overlapping constructs without clearly distinguishing them conceptually. For example:

- Resilience is described as both a process and an outcome,

- well-being appears both as a component and endpoint of resilience,

- meaning making overlaps with post-traumatic growth.

This creates conceptual redundancy. Recommendation

- clearer operational distinctions,

- conceptual model,

- explicit explanation of relationships among constructs.

7. Research design: is good and was indicated.

8. Clearly providing research questions and/or purpose: is good, as it has been indicated in the aims, but some journals may require hypotheses.

9. Choice of research method: Is good. But can you improve on the following:

a. Interview Guide: You mention 15 interview questions, but do not provide:

i. Examples of key questions.

ii. probing techniques.

b. Reflexive Depth

Although positionality is briefly acknowledged, reflexivity is superficial.

You did not discuss:

i. How their professional roles influenced interviews

ii. possible interviewer bias.

iii. emotional responses during interviews.

Given the sensitivity of IPV research, reflexivity is crucial.

c. Data Saturation poorly explained

The manuscript states saturation was achieved, but does not explain:

i. How saturation was determined

ii. Which themes became repetitive

iii. whether code saturation or meaning saturation was used.

10. Ethical Procedures Need Expansion: Fairly written. The ethics section is minimal. Important issues are insufficiently addressed:

i. participant safety protocols.

ii. trauma-informed interviewing.

iii. psychological support after interviews.

iv. confidentiality safeguards.

This is especially important in IPV research.

11. Appropriateness of procedures chosen for data collection and analysis: Good. Cultural Context Is Underdeveloped. Although the study is situated in southeastern Türkiye, the sociocultural context is insufficiently analyzed. The manuscript briefly mentions patriarchy, gender inequality, arranged marriage, and stigma. However, it did not deeply examine regional conservatism, ethnic/cultural influences, religion-politics intersections, family honor dynamics, collectivist pressures, rural versus urban differences. This is a missed opportunity.

12. Relevance of data obtained in view of the purpose of the research: Fair. This can be improved by analyzing differences by: Education, employment, marital status, age, motherhood, socioeconomic class. Moreso the sociodemographic variables were part of your research question.

13. Discussion of the results and their significance: Fairly written.

You can improve this section:

A) a. What new theoretical understanding emerges,

b. How Turkish sociocultural dynamics uniquely shape resilience.

c. Explain what is uniquely Turkish or region-specific.

B) Reduce some repetitions of findings in the discussion.

C) What Stronger Analytical Discussion Usually Includes:

A stronger discussion would:

• compare findings with conflicting studies,

• explain surprising results,

• discuss cultural/theoretical implications,

• identify tensions or contradictions,

• explain what new knowledge the study contributes.

For example:

• Why was religion especially important in this region?

• Why did women normalize violence initially?

• How does Turkish patriarchal culture shape resilience differently from Western findings?

• Did resilience emerge despite support systems rather than because of them?

14. Soundness of conclusions in relation to data presented: Good.

15. Limitation: well-written.

16. Implication/Recommendation: Well written.

17. Format of referencing: You have to follow what the journal indicates.

18. Figure/tables: Table 1 is absent.

19. In-text reference: well written

20. References: Good

# Editor report: Decision: Resilience among women survivors of intimate partner violence: Coping, meaning-making, and well-being — R0/PR5

**DOI:** 10.1017/gmh.2026.10262.pr5

**Affiliations:** London School of Hygiene & Tropical Medicine, United Kingdom of Great Britain and Northern Ireland

No accompanying comment.

# Reviewer report: Review: Resilience among women survivors of intimate partner violence: Coping, meaning-making, and well-being — R1/PR7

**DOI:** 10.1017/gmh.2026.10262.pr7

This version is clear and discuss in details.

# Reviewer report: Review: Resilience among women survivors of intimate partner violence: Coping, meaning-making, and well-being — R1/PR8

**DOI:** 10.1017/gmh.2026.10262.pr8

**Authors:** Asiya Muhammad

**Affiliations:** Clinical Services Department, Federal Neuro-Psychiatric Hospital Kaduna, Nigeria

Dear researchers,

I have enjoyed reading your paper and learnt a lot- thanks for your contribution to mental heath. I am looking forward to seeing the paper published.

# Editor report: Recommendation: Resilience among women survivors of intimate partner violence: Coping, meaning-making, and well-being — R1/PR9

**DOI:** 10.1017/gmh.2026.10262.pr9

**Affiliations:** Psychology, UNISA, South Africa

Dear Dr. Sakiz,

Thank you for your revised manuscript. We have reached a decision regarding

your submission. I am pleased to inform you that your manuscript has now

been accepted for publication.

The Editorial Office will contact you to finalise your manuscript. If you need any assistance, kindly contact the Editorial Office

Sincerely,

Dr. Limkile Mpofu

Cambridge Prisms: Global Mental Health

# Editor report: Decision: Resilience among women survivors of intimate partner violence: Coping, meaning-making, and well-being — R1/PR10

**DOI:** 10.1017/gmh.2026.10262.pr10

**Affiliations:** London School of Hygiene & Tropical Medicine, United Kingdom of Great Britain and Northern Ireland

No accompanying comment.
